# Acupuncture methods for allergic rhinitis: a systematic review and bayesian meta-analysis of randomized controlled trials

**DOI:** 10.1186/s13020-020-00389-9

**Published:** 2020-10-12

**Authors:** Zihan Yin, Guoyan Geng, Guixing Xu, Ling Zhao, Fanrong Liang

**Affiliations:** School of Acu-Mox and Tuina, Chengdu University of Traditional Chinese Medicine, 37 Shi Er Qiao Road, Chengdu, 610075 China

**Keywords:** Acupuncture, Allergic rhinitis, Systematic review, Network meta-analysis

## Abstract

**Background:**

Allergic rhinitis (AR) is a common symptomatic, inflammatory, and immunological disorder of nasal mucosa. Multiple clinical trials and systematic reviews have implicated acupuncture methods as potentially effective treatment strategies for AR, however, considering the great burden of AR, it is crucial to explore the most recent clinical evidence supporting acupuncture in AR. Besides, the methodologies reported in previous studies as well as those commonly applied during clinical practices greatly vary. Herein, we conducted network meta-analysis to compare the effectiveness of diverse acupuncture methods for AR treatment.

**Methods:**

We conducted a literature search for relevant reports published from inception to 1 July 2020 in several scientific databases, including PubMed, Embase, Cochrane library, Web of Science, CNKI, WF, VIP, CBM, AMED as well as related registration platforms. Primary outcomes as reported in the identified studies were assessed using nasal symptoms. All Meta-analyses were performed with RevMan, ADDIS, and STATA software. To ensure consistency among our reviewers, the intra-class correlation coefficient was used.

**Results:**

Exactly 39 studies with 3433 participants were covered in this meta-analysis. The meta-analysis demonstrated that all acupuncture types were superior to sham acupuncture in terms of total nasal symptom score and rhinoconjunctivitis quality of life questionnaire. Moxibustion was recommended as the most effective intervention as it reduced nasal symptoms in 6 treatments. On the other hand, manual acupuncture plus conventional medicine was recommended as the most effective intervention in improving the quality of life in 9 treatments. Notably, moxibustion was recommended as the most effective intervention that changed the content of IgE in 9 treatments. Moreover, adverse events of these interventions were acceptable.

**Conclusion:**

Our findings revealed that all acupuncture methods are effective and safe for AR. Moreover, either moxibustion or manual acupuncture plus conventional medicine are potentially the most effective treatment strategies for AR. Based on these findings, it is evident that acupuncture therapy is not inferior to pharmacologic therapy. Therefore, for AR patients who are either unresponsive to conventional medicine or are intolerant to adverse events, acupuncture therapy should be administered. However, the quality of these included trials was mainly ranked as moderate quality, we recommend additional well-designed RCTs with larger sample sizes to validate these findings.

## Background

Allergic rhinitis (AR), an inflammation of the nasal mucous membranes, is induced through immunoglobulin E (IgE)-mediated responses to aeroallergens [1, 2]. The condition is manifested by sudden and recurrent nasal congestion, itchiness, sneezing, and runny nose [3]. AR is prevalent across the globe, [4–6] it is estimated that nearly 10–20% of the global population suffers from the disease [7], especially in China [8–10]. In the US, the disease reportedly affects up to 60 million people annually, with self-reported rates at 10–30% in adults and 40% in children [7]. In addition, the US reports annual direct health expenditures range of between $2 and 5 billion. AR negatively affects the quality of life and results in reduced work productivity, [11–14] thus is considered a serious threat to the way of life globally.

Treatment therapies for AR and/or asthma comprise the use of several conventional medicines (CM) such as antihistamines, bronchodilators, and glucocorticoids [15]. However, the efficacy of these medications is not significant. Consequently, safer and more effective therapies are required to ensure effective management of the conditions. Apart from the aforementioned pharmacological methods, guidelines for clinical practice for AR stipulate that clinicians can administer non-pharmaceutical therapy treatment to patients [16, 17] and that 1 in every 5 people will always choose acupuncture [18]. Numerous guidelines have recommended acupuncture for managing AR [13, 18, 19]. Of note, this therapy has a long history in China and has successfully been used to manage AR symptoms [20–22]. Some studies reported that acupuncture achieved similar medicinal efficacy in treating moderate/severe AR, and the approach was safe without any apparent adverse reactions.[23, 24]. Further, numerous studies have shown that neuroendocrine immunity (NEI) plays a important role in AR [25, 26] and acupuncture may affect the function of the NEI system to treat AR. [13, 27–29].

In the recent past, an increasing number and size of random controlled trials (RCTs) for AR have been conducted with several systematic reviews [20, 21, 30–33], confirming the efficacy and safety of acupuncture. However, these studies have only focused on a single acupuncture therapy against a control treatment for AR. Additionally, the systematic reviews only considered the cumulative effects of all the acupuncture treatments. Most articles only report on the evidence obtained upon comparing acupuncture methods with drugs or placebo acupuncture methods, however, they do not compare results across all existing acupuncture methods included in systematic reviews, such as manual acupuncture (MA), moxibustion (Mox), electronic acupuncture (EA), warm acupuncture (WA), acupuncture-moxibustion (AM), and fire acupuncture (FA). There is a need, therefore, to identify the optimal acupuncture methods for AR treatment.

Using the Bayesian network meta-analysis (NMA), we compared and rank the efficacy and safety of all acupuncture therapies adopted in AR treatment. Our findings provide credible evidence for the use of acupuncture therapies and elucidate the current controversies surrounding the approaches for their effective application in clinical operations as well as guiding health policies.

## Methods

The systematic review is registered on PROSPERO, under the number CRD42020156200, in accordance with the Preferred Reporting Items for Systematic Reviews and Meta-Analyses (PRISMA-NMA) [34], and the accompanying checklist.

### Eligibility criteria and exclusion criteria

#### Types of studies

All articles reporting on RCTs and published in English/Chinese, without any regional and publication restrictions, were included. The first period of the randomized cross-over trials was applied. Conversely, non-randomized clinical studies, quasi and cluster RCTs, case reports as well as studies where no data are available were excluded.

#### Types of participants

All patients, either male or female, and across all ages, who were diagnosed with AR were included. Similarly, participants with all types of AR (including intermittent AR and persistent AR) regardless of their gender, etiology, ethnic group, severity, or diagnosed with specific criteria (such as mentioning any one of the criteria for the diagnosis of AR) met the inclusion criteria. Patients that diagnosed rhinitis which cannot be clearly attributed to allergic disorder would be excluded.

#### Types of intervention

Here, we included studies reporting on the use of acupuncture as monotherapy or additional therapies. The expected acupuncture approaches include MA, Mox, EA, AM, WA, and FA among others. Moreover, articles describing combinations of these acupuncture methods with CM, regardless of needling technique or acupoint selections, were included, the primary acupuncture methods were mentioned. Besides, we did not include acupoint embedding, application, and injection, as well as bee venom acupuncture and other treatments because they used related drugs. Studies that integrated acupuncture with blood-letting therapy, cupping, and herbal medicine, were excluded from the analysis.

#### Type of control group

Different acupuncture therapies formed the basis for the control group and included both a placebo group (sham acupuncture (SA)) as well as a conventional-based medicine group.

#### Types of outcome measures

We included studies that covered one or more of the below-highlighted outcomes. Our systematic review primarily aimed to compare and rank the efficacy and safety of all acupuncture methods used in AR treatment. Based on this, the primary acceptable outcomes were nasal symptoms of AR, as numerous previous studies have reported the use of total nasal symptom score (TNSS). Secondary outcomes were analyzed as follows: (1) Quality of life was measured using rhinoconjunctivitis quality of life questionnaire (RQLQ); (2) IgE concentration was used to assess serum allergen-specific IgE; (3) Safety measures were assessed by describing adverse events (AEs) directly connected to the intervention. Outcome measures that were not relevant to AR would be excluded.

### Search strategy

To retrieve relevant articles, we searched the databases including CBM, CNKI, WF, VIP, Web of Science (WOS), Embase, PubMed, and Cochrane Library, from their inception to 1st July 2020. Additionally, other resources such as clinical trial registries (WHO ICTRP, Clinical Trials, and ChiCTR) and Allied and Complementary Medicine Database (AMED) were used to reduce publication bias in data. Additional trials, including relevant systematic reviews and meta-analyses, were identified according to the list of all identified publications. Since different databases required different retrieval methods, our search terms covered three groups: (1) Clinical conditions namely AR, Allergic Rhinitis, and, rhinallergosis; (2) acupuncture methods such as acupuncture therapy, manual acupuncture, moxibustion, electroacupuncture, and fire acupuncture, etc.; (3) study types such as randomized controlled trials. The mode of retrieval used was dependent on subject terms and free words, and these terms distinguished between published studies. We used “and”, “or” to connect the name of terms. According to PubMed’s search strategy (Table 1), Chinese and English retrieval modes were expected to be similar.

**Table 1:** Search strategy (through PubMed)

Search query
1. Search “Rhinitis, Allergic”[Mesh] OR “Allergic Rhinitis”[tiab] OR “rhinallergosis”[tiab]
2. Search (“clinical”[tiab] AND “trial”[tiab]) OR “clinical trial”[Publication Type] OR “random*”[Title/Abstract] OR “clinical trials as topic”[MeSH Terms] OR “random allocation”[MeSH Terms] OR “therapeutic use”[MeSH Subheading]
3. Search 1 AND 2
4. Search “Acupuncture Therapy”[Mesh] OR “Acupuncture”[Mesh] OR “Moxibustion”[Mesh] OR “acupuncture”[tiab] OR “electroacupuncture”[tiab] OR “acupuncture-moxibustion”[tiab] OR “meridian*”[tiab] OR “acupoint”[tiab]OR “acupuncture points”[tiab] OR “acupressure-acupuncture therapy”[tiab] OR “warm needling”[tiab]OR “moxa needle”[tiab] OR “acupuncture plus moxibustion”[tiab] OR “moxibustion with warming needle”[tiab] OR “auricular acupuncture”[tiab] OR “auricular needle”[tiab] OR “ear acupuncture”[tiab] OR “moxibustion”[tiab] OR “electronic acupuncture”[tiab] OR “fire acupuncture”[tiab] OR “electronic acupuncturetranscutaneous electrical acupoint stimulation”[tiab]
5. Search 3 AND 4
6. Search 5 AND “English”[lang]

### Study selection and data extraction

First, all reviewers were professionally trained to master the review process. To select the studies, the reviewers first read the title/abstract of the review to identify duplicate studies, then, they uploaded eligible articles to a database built using NoteExpress V.3.0. Further, the two reviewers (GG and GX) independently schemed through the titles, abstracts, and keywords. The two reviewers solved any disagreements through a consensus, following a discussion. However, whenever the discussion was conflicting, a third reviewer (LZ) would help in making a final decision.

The aforementioned reviewers independently extracted data using a standardized eligibility form, and in case of a disagreement, a third reviewer (LZ) came in. Any missing information was obtained by contacting the corresponding author of the specific article. General data from the selected studies including, the name of the first author, year of publication, country, study design, sample size, intervention group, control group, outcome, results, were extracted and recorded into an Excel sheet. Also, the reviewers independently measured the risk of bias in the included studies following the guidelines of the Cochrane Handbook V.5.3.0. In case of insufficient or ambiguous data, one reviewer contacted the corresponding author of the articles, requesting for additional details. However, if the details were not available, a description was added to the final report. A summary of the selection procedures is outlined using the PRISMA flow chart.

### Study quality assessment

Here, 2 reviewers evaluated the risk of bias in all included RCTs using the Cochrane Collaboration Risk of Bias Tool [35]. This approach comprised sequence generation, allocation concealment, blinding of participants and outcome assessors, incomplete outcome data, selective outcome reporting, as well as other sources of bias. The findings were used to rank risk levels as low, unclear, or high. In case of any disagreement, the third reviewer (LZ) was consulted to generate a consensus. Review Manager (RevMan, version 5.3, the Nordic Cochrane Center, the Cochrane Collaboration, 2012 Copenhagen, Denmark) was used to generate the figure highlighting the risk of bias.

### Pairwise meta-analysis

Revman was used to analyze the data. Pre-post differences were used as outcome indicators for each included study. Further, 3-arm RCTs were separated into two arms for all possible combinations in the meta-analysis. The fixed-effects model utilized the Mantel–Haenszel procedure, otherwise, the random-effects model adopted by Der Simonian-Laired procedure was used. The I^2^ statistic and *p* value were used to identify and measure the heterogeneity among the studies. All data were analyzed with a 95% confidence interval (CI). For continuous data, the standard mean differences (SMD) were used. According to the Cochrane Handbook, when *p* > 0.05, I^2^ < 50%, we considered that no heterogeneity existed.

### Network meta-analysis

To compare the effects of different acupuncture treatments, a Bayesian network analysis was performed [36] using the Aggregate Data Drug Information System (ADDIS V.1.16.8, Drugis, Groningen, NL), with Markov Chain Monte Carlo (MCMC) method [37]. The parameters were set at 4 chains for simulation, while the simulation iterations were set to 50,000. First, we performed 20,000 adjustment iterations to eliminate the effect of the initial value, then, integrated indirect and direct evidence from all the RCTs according to the node splitting method. Meanwhile, STATA software Version.15.0 (Stata Corp LP, College Station, Texas, USA) was used to generate plots of the network meta-analysis and compare each outcome. Finally, we generated figure ranking probabilities for all the interventions, after which local inconsistency was assessed using the node-splitting method. Generally, all nodes showed P-values greater than 0.05 in inconsistency tests, implying that no significant statistical difference existed between direct and indirect comparisons. Potential scale reduced factor (PSRF) reflected the convergence of the model, with a PSRF of 0 and 100% indicating the worst and best treatments, respectively.

### Publication bias

We performed a funnel plot indicating digital-based modeling of the results to eliminate reporting data with bias.

### Assessing reviewer agreement

We performed the intra-class correlation coefficient (ICC) to evaluate the consistency of two reviewers. Briefly, the two authors independently evaluated the quality of RCTs. The composite ICC score value was 0.93.

## Results

### Study selection

**Fig. 1:**
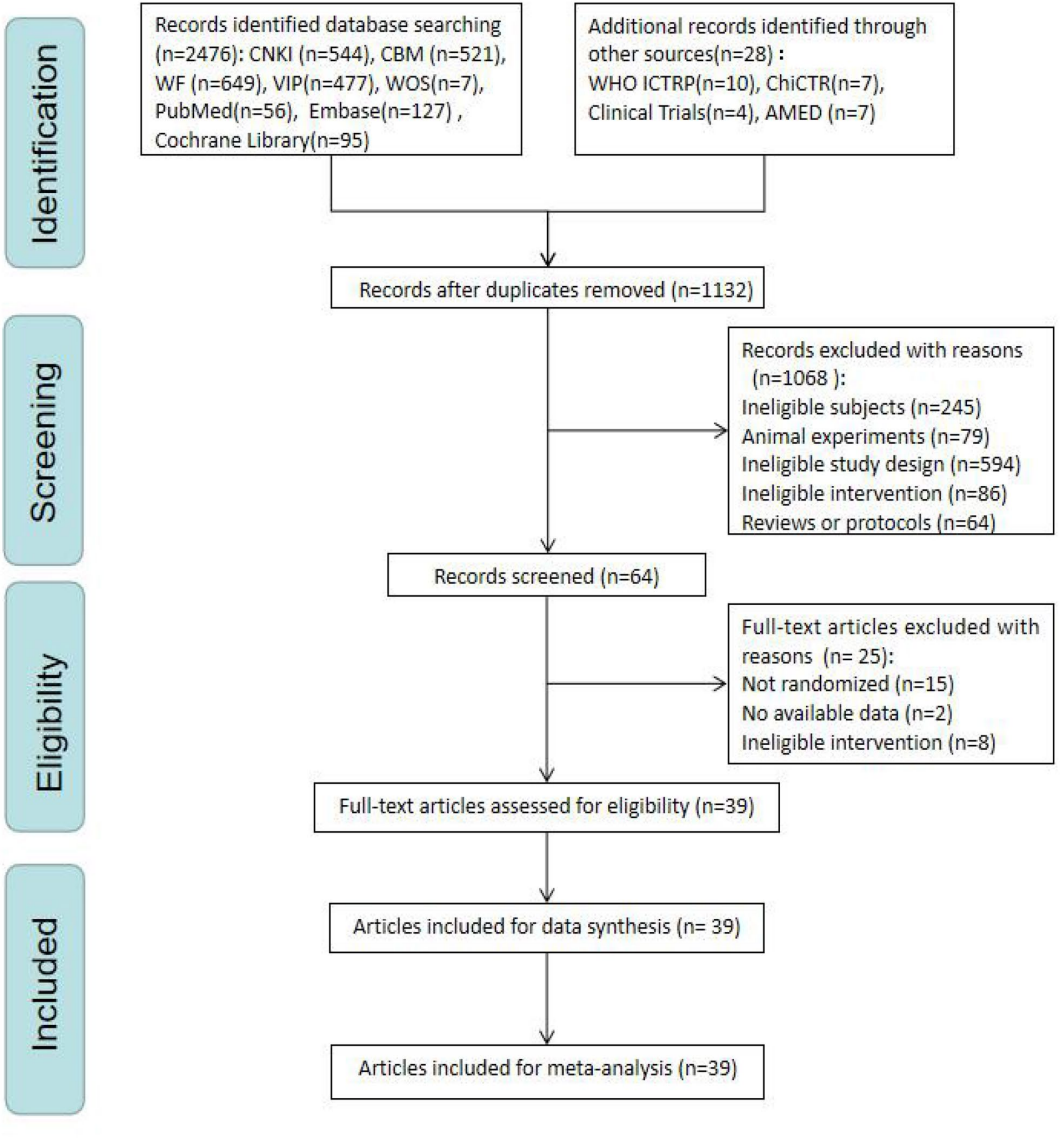
The PRISMA flow chart of selection process

After the primary search process, we identified 3004 potentially relevant studies from these databases. After eliminating 1132 duplicates, 1872 articles were retained, from which only 64 remained after reading through the titles and abstracts. When we did a full-text assessment, 25 articles were excluded. Eventually, a total of 39 RCTs were included [38–76] in this systematic review (Fig. 1).

### Study characteristics

In total, 39 studies were included in the final Bayesian meta-analysis, 37 RCTs [38–71, 73, 75, 76] were published in Chinese while and 2 RCTs [72, 74] were written in English. All the 39 articles were published between 2006 and 2020, with 3433 participants. The interventions in these studies included MA, EA, WA, AM, FA, Mox, SA, CM, and the combinations between these methods or with CM. Besides, the mean patient age of 26 to 45 years (except that 7 RCTs didn’t specify clear age [38, 48, 52, 64, 69, 75, 76]). The sample size of the included RCTs ranged from 37 to 422. Group allocation was primarily performed using a 1:1 allocation ratio. And the ratio of male to female is basically the similar. The selection of acupoint in the included acupuncture treatments was diverse, the main choices included Xinwu point (Sphenopalatine Ganglion), Yingxiang (LI 20), Yintang (DU 29), Shangyingxiang point, Feishu (BL 13), Dazhui (DU 14), Hegu (LI4). In these studies, treatment duration ranged from 4 to 8 weeks. A reduction in the RQLQ score was, in most cases the mentioned outcome measures. Detailed information and findings of all included studies are displayed in Table 2.

**Table 2:** Main characteristics of included RCTs

Study	Country	Sample size	Allocation ratio	Age	Gender (M:F)	(A)	(B)	(C)	Duration of treatment	Efficacy and safety criteria	Main results
						Treatment Group	Control Group I	Control Group II			
Hou 2020 [38]	China	60	1:1	/	/	MA on Sibai (ST 2) with stimulation 1 × /day	CM (10 mg/ day dose of loratadine as a 10 mg capsule)	/	2 weeks	1. Reduction of Ig E	1. A > B
Wu 2020 [39]	China	80	1:1	A: 32.5 ± 1.8 B: 31.1 ± 2.3	A: (20:20) B: (21:19)	MA on Xinwu with stimulation 2 × /week + (B)	CM (Two arms: (1) 10 mg/ day dose of loratadine as a 10 mg capsule (2) 200 ug/day of Mometasone Furoate Aqueous Nasal Spray as 4 50 ug capsules)	/	4 weeks	1. Reduction of Ig E 2. Reduction of RQLQ	1. A > B 2. A > B
Zhang 2020 [40]	China	180	1:1	A: 42.37 ± 16.14 B: 39.27 ± 15.21	A: (31:59) B: (37:53)	Mox on Dazhui (DU 14), Yintang (DU 29), Feishu (BL 13), with stimulation 5 × /week	CM (Twice of Budesonide Nasal Spray)	/	4 weeks	1. Reduction of RQLQ	1. A > B
Sun 2020 [41]	China	210	1:1	A: 38.25 ± 8.84 B: 36.52 ± 9.86	A: (58:42) B: (58:42)	WA on Yingxiang (LI 20), Yintang (DU 29), Baihui (DU 20), Hegu (LI 4), Guanyuan (RN 4), Zusanli (ST 36), Dazhui(DU 14), Fengchi (GB 20), Jiaji (EX B2), Ganshu (BL 18), Shenshu (BL 23) with stimulation 4 × /week	CM (Two arms: (1) 8.8 mg/ day dose of loratadine as a 8.8 mg capsule (2) 50 ug/day of Fluticasone Propionate Nasal Spray as 2 50 ug capsules)	/	4 weeks	1. Reduction of Ig E 2. Adverse Events	1. A > B 2. A > B
Song 2020 [42]	China	64	1:1	A: 43.7 ± 13.9 B: 44.6 ± 13.4	A: (17:15) B: (19:13)	MA on Xinwu with stimulation. 1 × /week	CM (Two arms: (1) 10 mg/ day dose of loratadine as a 10 mg capsule (2) Once/day of Budesonide Nasal Spray as 2capsules)	/	4 weeks	1. Reduction of Ig E	1. A > B
Wang 2020 [43]	China	80	1:1	A: 29 ± 12 B: 28 ± 12	A: (12:28) B: (15:25)	Mox on Guanyuan (RN 4), Yintang (DU 29), Feishu (BL 13), with stimulation 5 × /week	CM (Two arms: (1) 5 mg/ day dose of loratadine as a 5 mg capsule (2) Once/day of Triamcinolone Acetonide Nasal Spray as 2capsules)	/	3 weeks	1. Reduction of Ig E 2. Reduction of RQLQ	1. A > B 2. A > B
Zhang 2019 [44]	China	60	1:1	A: 33.93 ± 10.19 B: 34.73 ± 11.15	A: (13:17) B: (13:17)	WA on Yingxiang(LI 20), Yintang (DU 29), Hegu (LI 4), Zusanli (ST 36), with stimulation 2 × /week	CM (Once/day of Mometasone Furoate Aqueous Nasal Spray as 1–2 capsules)	/	4 weeks	1. Reduction of RQLQ 2. Adverse Events	1. A > B 2. A < B
Gao 2019 [45]	China	98	1:1	A: 41.41 ± 9.99 B: 41.08 ± 12.00	A: (18:31) B: (20:29)	WA on Yingxiang (LI 20), Yintang (DU 29), Sibai (ST 2), Dazhui (DU 14), Hegu (LI 4), Shangxing (DU 23), Chizhe (LU 5), Shangyingxiang, with stimulation 3 × /week	CM (10 mg/ day dose of loratadine as a 10 mg capsule)	/	4 weeks	1. Reduction of TNSS 2. Reduction of RQLQ 3. Adverse Events	1. A > B 2. A > B 3. A > B
Liao 2019 [46]	China	64	1:1	A: 35.82 ± 1.61 B: 36.36 ± 1.57	A: (19:13) B: (17:15)	WA on Yingxiang (LI 20), Yintang (DU 29), Sibai (ST 2), Dazhui(DU 14), Hegu (LI 4), Shangxing (DU 23), Chizhe (LU 5), Lieque (LU 7), Shangyingxiang, with stimulation 3 × /week	CM (10 mg/ day dose of loratadine as a 10 mg capsule)	/	4 weeks	1. Reduction of RQLQ	1. A > B
Li 2019 [47]	China	114	1:1:1	A: 36.69 ± 2.19 B: 38.35 ± 2.30 C: 36.35 ± 2.06	A: (20:17) B: (19:19) C: (19:18)	MA (Acupuncture on Neiyingxiang with stimulation 1 × /day)	CM (10 mg/ day dose of loratadine as a 10 mg capsule)	(A) + (B)	2 weeks	1. Reduction of TNSS 2. Reduction of RQLQ	1. C > A > B 2. C > A > B
Wang 2019 [48]	China	200	1:1	/	/	WA on Yingxiang (LI 20), Shenshu (BL 23), Fengchi (GB 20), Hegu (LI 4), Zusanli (ST 36), Feishu (BL 13), Shangyingxiang, with stimulation 5 × /week	CM (10 mg/ day dose of loratadine as a 10 mg capsule)	/	2 weeks	1. Reduction of Ig E	1. A > B
Lu 2018 [49]	China	62	1:1	A: 39.00 ± 11.29 B: 39.40 ± 11.56	A: (20:12) B: (17:13)	MA on Guanyuan (RN 4), Qihai (RN 6), Zhongwan (RN 12), Xiawan (RN 10), Shangqu (KI 17) etc. with stimulation 3 × /week	CM (Twice/day of Budesonide Nasal Spray as 4capsules)	/	4 weeks	1. Reduction of TNSS	1. A > B
Zhao 2018 [50]	China	61	1:1	A: 39.19 ± 11.25 B: 39.40 ± 11.56	A: (14:17) B: (17:13)	MA on Guanyuan (RN 4), Qihai (RN 6), Zhongwan (RN 12), Xiawan (RN 10), Shangqu (KI 17) etc. with stimulation 3 × /week	CM (Twice/day of Budesonide Nasal Spray as 4capsules)	/	4 weeks	1. Reduction of TNSS 2. Reduction of RQLQ 3. Adverse Events	1. A > B 2. A > B 3. A > B
Yuan 2018 [51]	China	58	1:1	A: 34.41 ± 9.59 B: 38.52 ± 10.73	A: (10:19) B: (16:13)	WA on Yingxiang (LI 20), Yintang (DU 29), Hegu (LI 4), Zusanli (ST 36), with stimulation 7 × /2 weeks	CM (1/day of Budesonide Nasal Spray)	/	2 weeks	1. Reduction of RQLQ	1. A > B
Fang 2018 [52]	China	50	1:1	/	29:21	AM on Xinwu with stimulation 2 × /week + (B)	CM (Two arms: (1) 8 mg/ day dose of loratadine as a 8 mg capsule (2)Budesonide Nasal Spray as 2 64 ug capsules)	/	4 weeks	1. Reduction of Ig E 2. Reduction of RQLQ	1. A > B 2. A > B
Wen 2018 [53]	China	59	1:1	A: 35.45 ± 9.92 B: 34.40 ± 10.25	A: (12:17) B: (14:16)	WA on Qihai (RN 6), Zhongwan (RN 12), Zusanli (ST 36), Yinglingquan (SP 9), with stimulation 1 × /day	CM (Two arms: (1) 10 mg/ day dose of loratadine as a 10 mg capsule (2) Once/day of Triamcinolone Acetonide Nasal Spray as 4capsules)	/	4 weeks	1. Reduction of Ig E 2. Reduction of RQLQ 3. Adverse Events	1. A > B 2. A > B 3. A = B
Li 2018a [54]	China	90	1:1	A: 35.97 ± 7.47 B: 36.09 ± 7.52	A: (25:20) B: (27:18)	Mox on Yintang (DU 29), Feishu (BL 13), Dazhui(DU 14), with stimulation 1 × /day + (B)	CM (Two arms: (1) 10 mg/ day dose of loratadine as a 10 mg capsule (2)Budesonide Nasal Spray as 2 200 ug capsules)	/	4 weeks	1. Reduction of TNSS 2. Reduction of RQLQ	1. A > B 2. A > B
Li 2018b [55]	China	126	1:1	A: 35.42 ± 7.51 B: 36.90 ± 7.45	A: (35:28) B: (33:30)	WA on Quchi (LI 11), Hegu (LI 4), Feishu (BL 13), Dazhui(DU 14), Shangxing (DU 23), Zusanli (ST 36), with stimulation 1 × /day	CM (Once a day of loratadine)	/	10 days	1. Reduction of Ig E 2. Reduction of RQLQ 3. Adverse Events	1. A > B 2. A > B 3. A = B
Li 2018c [56]	China	54	1:1	A: 41.1 ± 10.7 B: 41.0 ± 9.5	A: (14:13) B: (12:15)	MA on Xinwu with stimulation. 2 × /week + (B)	CM (5 mg/ day dose of loratadine as a 5 mg capsule)	/	4 weeks	1. Reduction of TNSS	1. A > B
Zheng 2017 [57]	China	50	1:1	A: 41.95 ± 11.10 B: 39.71 ± 11.82	A: (7:15) B: (11:13)	WA on Yingxiang(LI 20),Yintang (DU 29), Sibai (ST 2), Shangyingxiang, Shangxing (DU 23), Hegu (LI 4), Dazhui(DU 14), Chizhe (LU 5), Lieque (LU 7), with stimulation 3 × /week	CM (10 mg/ day dose of loratadine as a 10 mg capsule)	/	4 weeks	1. Reduction of RQLQ 2. Adverse Events	1. A > B 2. A = B
Cao 2017 [58]	China	86	1:1	A: 36.2 ± 4.8 B: 37.1 ± 4.6	A: (27:16) B: (25:18)	AM on Yingxiang(LI 20), Yintang (DU 29), Hegu (LI 4), Dazhui(DU 14), Fengchi (GB 20), Lieque (LU 7), with stimulation 1 × /day	MA on Yingxiang(LI 20), Yintang (DU 29), Hegu (LI 4), Dazhui(DU 14), Fengchi (GB 20), Lieque (LU 7), Shangyintang, Feishu (BL 13) with stimulation with stimulation 1 × /day	/	30 days	1. Reduction of Ig E	1. A > B
Li 2016a [59]	China	27	1:1	A: 43.75 ± 12.67 B: 34.38 ± 12.93	A: (5:7) B: (7:6)	WA on Yingxiang (LI 20), Yintang (DU 29), Sibai (ST 2), Dazhui(DU 14), Hegu (LI 4), Shangxing (DU 23), Chizhe (LU 5), Shangyingxiang, Lieque (LU 7), with stimulation. 3 × /week	CM (10 mg/ day dose of loratadine as a 10 mg capsule)	/	4 weeks	1. Reduction of TNSS 2. Reduction of RQLQ	1. A > B 2. A > B
Liu 2016 [60]	China	60	1:1:1	A: 33.75 ± 10.82 B: 32.50 ± 9.79 C: 33.40 ± 11.11	A: (7:13) B: (4:16) C: (9:11)	WA on Quchi (LI 11), Hegu (LI 4), Feishu (BL 13), Dazhui(DU 14), Yingxiang (LI 20), Zusanli (ST 36), with stimulation 1 × /day	MA on Quchi (LI 11), Hegu (LI 4), Feishu (BL 13), Dazhui(DU 14), Yingxiang (LI 20), Zusanli (ST 36), with stimulation 1 × /day	AM on Quchi (LI 11), Hegu (LI 4), Feishu (BL 13), Dazhui(DU 14), Yingxiang (LI 20), Zusanli (ST 36), with stimulation 1 × /day	17 days	1. Reduction of Ig E 2. Reduction of RQLQ 3. Adverse Events	1. A > B > C 2. A > C > B 3. A = B = C
Li 2016b [61]	China	60	1:1	A: 40.20 ± 12.52 B: 42.00 ± 10.87	A: (7:23) B: (15:15)	Mox on Yintang (DU 29), Dazhui(DU 14), with stimulation 3 × /week	CM (10 mg/ day dose of cetirizine hydrochloride as a 10 mg capsule)	/	4 weeks	1. Reduction of TNSS 2. Adverse Events	1. A > B 2. A = B
Jin 2016 [62]	China	70	1:1	A: 36.45 ± 6.96 B: 35.40 ± 9.23	A: (19:12) B: (18:12)	Mox on Feishu (BL 13), Zhongfu (LU 1), with stimulation 3 × /week	CM (10 mg/ day dose of loratadine as a 10 mg capsule)	/	2 weeks	1. Reduction of TNSS 2. Reduction of RQLQ	1. A > B 2. A < B
Chen 2016 [63]	China	60	1:1	A: 33 ± 8 B: 35 ± 10	A: (18:12) B: (14:16)	MA on Xinwu with stimulation 2 × /week + (B)	CM (Two arms: (1) 10 mg/ day dose of loratadine as a 10 mg capsule (2)Budesonide Nasal Spray as 1 200 ug capsules)	/	4 weeks	1. Reduction of Ig E 2. Reduction of RQLQ	1. A > B 2. A > B
Yu 2015 [64]	China	64	1:1	/	A: (18:14) B: (17:15)	MA on Xinwu, Yingxiang (LI 20), Yintang (DU 29), Hegu (LI 4), Baihui (DU 20), Lieque (LU 7), Taichong (LR 3) with stimulation 2 × /week	CM (10 mg/ day dose of cetirizine hydrochloride as a 10 mg capsule)	/	5 weeks	1. Reduction of TNSS 2. Reduction of RQLQ 3. Adverse Events	1. A > B 2. A > B 3. A = B
Chen 2015 [65]	China	66	1:1	A: 44 ± 9 B: 40 ± 11	A: (17:17) B: (14:18)	MA on Yingxiang (LI 20), Yintang (DU 29), Hegu (LI 4), Baihui (DU 20), Taichong (LR 3), Shenshu (BL 23), Feishu (BL 13), Ganshu (BL 18), Pishu (BL 20), Dazhui (DU 14) with stimulation 3 × /week	CM (10 mg/ day dose of cetirizine hydrochloride as a 10 mg capsule)	/	4 weeks	1. Reduction of TNSS 2. Reduction of RQLQ 3. Adverse Events	1. A > B 2. A > B 3. A = B
He 2014 [66]	China	60	1:1	A: 31.31 ± 13.40 B: 35.22 ± 14.60	A: (12:18) B: (15:15)	EA on Yingxiang (LI 20),Yintang (DU 29), Fengchi (GB 20), Shangyingxiang, with stimulation 1 × /2 days	CM (10 mg/ day dose of cetirizine hydrochloride as a 10 mg capsule)	/	40 days	1. Reduction of RQLQ 2. Adverse Events	1. A > B 2. A = B
Huang 2014 [67]	China	90	1:1:1	A: 26.4 ± 1.71 B: 28.2 ± 1.21 C: 27.3 ± 0.86	A: (16:14) B: (13:17) C: (17:13)	MA on Yingxiang (LI 20),Yintang (DU 29), Hegu (LI 4), Shangyingxiang with stimulation 3 × /week	FA on Tongtian (BL 7), Dazhui (DU 14) with stimulation. 3 × /week	(A) + (B)	4 weeks	1. Reduction of Ig E	1. B > C > A
Si 2014 [68]	China	60	1:1	A: 45.63 ± 2.71 B: 45.86 ± 2.28	A: (11:19) B: (12:18)	WA on Yingxiang (LI 20), Yintang (DU 29), Hegu (LI 4), Fengchi (GB 20), Zusanli (ST 36), Shangxing (DU 23), Waiguan (SJ 5), Shangyingxiang with stimulation 3 × /week	EA on Yingxiang (LI 20), Yintang (DU 29), Hegu (LI 4), Fengchi (GB 20), Zusanli (ST 36), Shangxing (DU 23), Waiguan (SJ 5), Shangyingxiang with stimulation 3 × /week	/	3–4 weeks	1. Reduction of Ig E 2. Adverse Events	1. A > B 2. A = B
Zhang 2013 [69]	China	64	1:1	/	A: (12:18) B: (15:17)	MA on Xinwu, Yingxiang (LI 20), Feishu (BL 13), Lieque (LU 7), Fengchi (GB 20), Zusanli (ST 36), Tongtian (BL 7), Cuanzhu (BL 2) with stimulation 3 × /week	CM (10 mg/ day dose of cetirizine hydrochloride as a 10 mg capsule)	/	4 weeks	1. Reduction of Ig E	1. A < B
Shi 2013 [70]	China	60	1:1	A: 33.60 ± 13.25 B: 35.13 ± 10.78	A: (13:17) B: (12:18)	MA on Yingxiang (LI 20), Yintang (DU 29), Hegu (LI 4), Baihui (DU 20), Taichong (LR 3), Shenshu (BL 23), Feishu (BL 13), Ganshu (BL 18), Pishu (BL 20), Dazhui (DU 14), Taichong (LR 3) with stimulation 3 × /week	CM (10 mg/ day dose of cetirizine hydrochloride as a 10 mg capsule)	/	4 weeks	1. Reduction of TNSS	1. A > B
Wang 2013 [71]	China	55	1:1	A: 40.19 ± 12.19 B: 38.68 ± 8.79	A: (11:16) B: (11:17)	MA on Yingxiang (LI 20), Yintang (DU 29), Hegu (LI 4), Baihui (DU 20), Taichong (LR 3), Shenshu (BL 23), Feishu (BL 13), Ganshu (BL 18), Pishu (BL 20), Dazhui (DU 14), Taichong (LR 3) with stimulation 3 × /week	CM (10 mg/ day dose of cetirizine hydrochloride as a 10 mg capsule)	/	4 weeks	1. Reduction of TNSS 2. Reduction of RQLQ 3. Adverse Events	1. A < B 2. A > B 3. B > A
Brinkhaus 2013 [72]	Germany	422	2:1:1	A: 33.4 ± 7.5 B: 33.0 ± 8.2 C: 32.2 ± 8.1	A: (82:130) B: (37:65) C: (52:56)	MA + (C)	SA + (C)	CM (2 doses of cetirizine hydrochloride)	8 weeks	1. Reduction of RQLQ	1. A > B > C
Lan 2010 [73]	China	36	1:1	A: 38.06 ± 11.62 B: 40.72 ± 10.69	A: (5:13) B: (4:14)	MA on Yingxiang (LI 20), Yintang (DU 29), Sibai (ST 2), Hegu (LI 4), Zusanli (ST 36), Shangxing (DU 23) with stimulation 3 × /week	SA with stimulation 3 × /week	/	4 weeks	1. Reduction of TNSS 2. Reduction of RQLQ 3. Adverse Events	1. A > B 2. A > B 3. B = A
Xue 2008 [74]	Australia	80	1:1	A: 42.5 ± 14.2 B: 44.2 ± 11.0	A: (20:22) B: (13:25)	MA on Yingxiang (LI 20), Yintang (DU 29), and Fengchi (GB 20) with stimulation 2 × /week	SA, with stimulation 2 × /week	/	8 weeks	1. Reduction of TNSS 2. Adverse Events	1. A > B 2. A = B
Li 2007 [75]	China	100	1:1	/	A: (21:29) B: (22:28)	EA on Xinwu, Shangyingxiang, Yingxiang (LI 20), Yintang (DU 29), Shenshu (BL 23), Feishu (BL 13), Pishu (BL 20) with stimulation 1 × /day	CM (30 mg/ day dose of cetirizine hydrochloride as 3 10 mg capsule)	/	34 days	1. Reduction of Ig E	1. A > B
Rao 2006 [76]	China	93	1:1	/	A: (26:21) B: (25:21)	MA on Yingxiang (LI 20), Yintang (DU 29), Shenshu (BL 23), Feishu (BL 13), Pishu (BL 20), Hegu (LI 4), Zusanli (ST 36), Fengchi (GB 20) with stimulation 1 × /day	CM (10 mg/ day dose of cetirizine hydrochloride as a 10 mg capsule)	/	4 weeks	1. Reduction of Ig E	1. A > B

*MA* manual acupuncture, *EA* electroacupuncture, *WA* warm acupuncture, *FA* fire acupuncture, *Mox* moxibustion, *AM* Acupuncture-Moxibustion, *SA* sham acupuncture, *CM* conventional medicine

### Study quality assessment

**Fig. 2:**
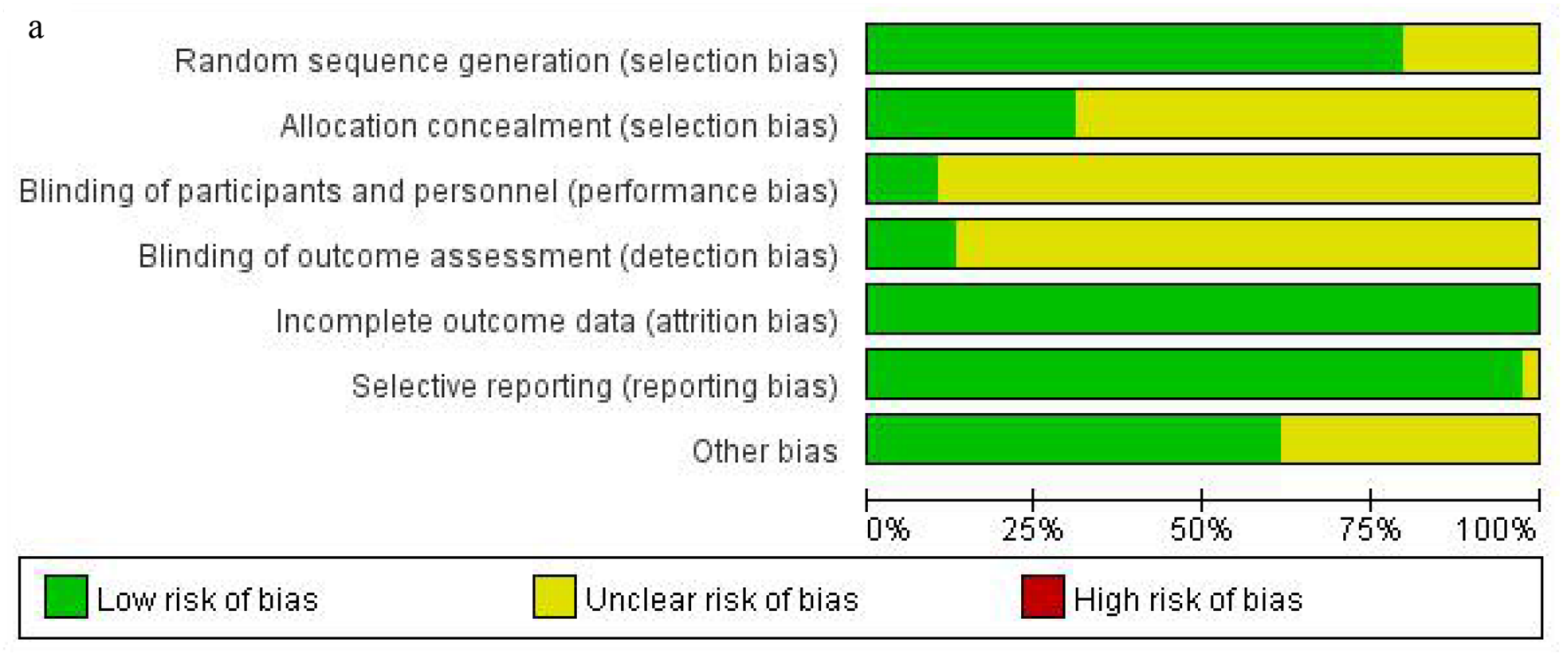

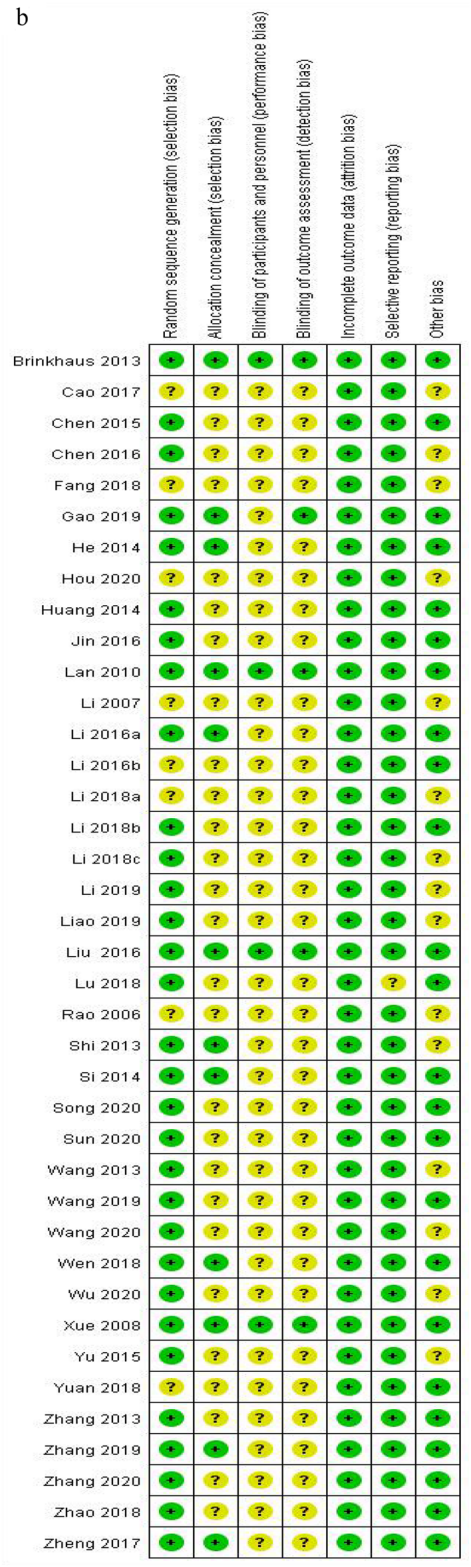
**a** Risk of bias graph; **b** Risk of bias summary

Herein, we assessed the risk of bias using the Cochrane Handbook Version 5.3. Meanwhile, the results were summarized and analyzed using RevMan 5.3. Overall, there were 4 [60, 72–74] high-quality RCTs in the included studies, but no case about high risk was reported. All RCTs were randomized, however, 8 trials [38, 51, 52, 54, 58, 61, 75, 76] did not use random sequence generation. Notably, 12 studies [44, 45, 53, 57, 59, 60, 68, 70–74] showed low risk of allocation concealment. The blinding method was performed in 5 studies [45, 60, 72–74], and all of them blinded outcome assessors, further, 4 RCTs [60, 72–74] showed that they blinded the participants. Data on the outcome from all the 5 studies were complete and ranked as low risk. In selective outcome reporting, 38 RCTs were evaluated as low risk, but one RCT [49] as unclear because of insufficient information. In the other bias, 14 trials [38, 39, 43, 46, 47, 52, 54, 56, 60, 63, 65, 69, 75, 76] were unclear as they lacked adequate information. The methodological quality assessment results for each included study are displayed in Fig. 2.

### Pairwise meta-analysis results

#### Reduction in TNSS

We performed 6 pairwise meta-analyses to compare the effectiveness of different acupuncture methods with CM. Detailed results are shown in Table 3. Mox and CM showed statistically significant differences (3 RCTs, SMD, 1.03; 95% CI 0.37 to 1.09); MA + CM was highly effective in improving TNSS compared to CM (1 RCTs, SMD, 3.30; 95% CI 2.29 to 4.31) and MA (1 RCTs, SMD, 1.47; 95% CI 0.74 to 2.21). No significant difference between MA and CM, WA and CM, MA and SA were noted.

**Table 3:** Pairwise meta-analysis of of reduction of TNSS

Comparison	Number	SMD (95% CI)	I^2^ (%)	*p*
MA VS CM	8	0.28 (− 0.25,0.81)	87	< 0.00001
WA VS CM	2	0.12 (− 0.19,1.33)	87	0.006
Mox VS CM	3	*1.03 (0.37,1.09)*	82	0.28
CM VS MA + CM	1	− *3.30 (*− *2.29,* − *4.31)*	–	–
MA VS MA + CM	1	− *1.47 (*− *0.74,* − *2.21)*	–	–
MA VS SA	2	1.05 (− 0.45,2.56)	92	0.0003

Italic values indicate significant difference

*MA* manual acupuncture, *WA* warm acupuncture, *Mox* moxibustion, *SA* sham acupuncture, *CM* conventional medicine

#### Reduction in RQLQ

Here, 13 pairwise meta-analyses were generated to compare the effectiveness of different acupuncture treatments with CM. Detailed results are shown in Table 4. MA + CM and MA showed statistically significant differences (1 RCT, SMD, − 1.92; 95% CI − 1.60 to − 0.24); CM and MA + CM displayed significant differences (3 RCTs, SMD, − 2.06; 95% CI − 2.42 to − 1.71); Statistically significant differences (1 RCT, SMD, 1.66; 95% CI 1.28 to 2.04) were reported between MA + CM and SA + CM; CM and SA + CM displayed significant differences (1 RCT, SMD, − 0.66; 95% CI − 1.06 to − 0.27); MA and SA displayed significant differences (1 RCT, SMD, 0.92; 95% CI 0.23 to 1.61); Statistically significant differences (8 RCTs, SMD, 1.71; 95% CI 0.87 to 2.56) were reported between WA and CM; CM and AM showed statistically significant differences (1 RCT, SMD, − 1.87; 95% CI − 2.57 to − 1.22). No significant difference was noted between MA and CM, MA and WA, MA and AM, WA and AM, Mox and CM, CM and EA.

**Table 4:** Pairwise meta-analysis of of reduction of RQLQ

Comparison	Number	SMD (95% CI)	I^2^ (%)	*p*
MA VS WA	1	− 0.53 (− 1.43, 0.36)	–	–
MA VS CM	6	0.79 (− 0.05, 1.63)	92	< 0.00001
MA VS MA + CM	1	− *1.92 (*− *1.60, *− *0.24)*	–	–
MA VS AM	1	− 0.13 (− 1.01, 0.74)	–	–
MA VS SA	1	*0.92 (0.23, 1.61)*	–	–
WA VS CM	8	*1.71 (0.87, 2.56)*	94	< 0.00001
WA VS AM	1	− 0.50 (− 0.39, 1.39)	–	–
Mox VS CM	4	0.14 (− 0.37, 0.64)	84	0.0003
CM VS MA + CM	3	− *2.06 (*− *2.42, *− *1.71)*	25	0.27
CM VS AM	1	− *1.87 (*− *2.57, *− *1.22)*	–	–
CM VS EA	1	− 0.13 (− 0.64, 0.37)	–	–
CM VS SA + CM	1	− *0.66 (*− *1.06, *− *0.27)*	–	–
MA + CM VS SA + CM	1	*1.66 (1.28, 2.04)*	–	–

Italic values indicate significant difference

*MA* manual acupuncture, *EA* electroacupuncture, *WA* warm acupuncture, *Mox* moxibustion, *AM* Acupuncture-Moxibustion, *SA* sham acupuncture, *CM* conventional medicine

#### Reduction in Ig E

We generated 13 pairwise meta-analyses to compare the effectiveness of different interventions. Detailed results are highlighted in Table 5. CM and MA + CM showed statistically significant differences (1 RCT, SMD, − 0.55; 95% CI − 1.00 to − 0.11). However, no significant difference was reported in others.

**Table 5:** Pairwise meta-analysis of of reduction of Ig E

Comparison	Number	SMD (95% CI)	I^2^ (%)	*p*
MA VS CM	5	0.06 (− 0.33, 0.46)	69	0.01
MA VS WA	1	− 0.73 (− 2.47, 1.02)	–	–
MA VS AM	2	− 0.31 (− 0.83, 0.21)	7	0.3
MA VS FA	1	− 0.15 (− 0.87, 0.57)	–	–
MA VS MA + FA	1	− 0.29 (− 1.01, 0.43)	–	–
WA VS CM	4	0.49 (− 0.25, 1.22)	94	< 0.00001
WA VS AM	1	1.34 (− 0.72, 3.40)	–	–
WA VS EA	1	− 0.1 (− 0.61, 0.41)	–	–
Mox VS CM	1	0.33 (− 0.11, 0.78)	–	–
CM VS MA + CM	1	− *0.55 (*− *1.00, *− *0.11)*	–	–
CM VS AM	1	− 0.55 (− 1.12, 0.01)	–	–
CM VS EA	1	− 0.36 (− 0.77, 0.02)	–	–
FA VS FA + AM	1	− 0.16 (− 0.87, 0.56)	–	–

Italic value indicates significant difference

*MA* manual acupuncture, *EA* electroacupuncture, *WA* warm acupuncture, *FA* fire acupuncture, *Mox* moxibustion, *AM* Acupuncture-Moxibustion, *CM* conventional medicine

### Network meta-analysis results

#### Network plot for different interventions

**Fig. 3:**
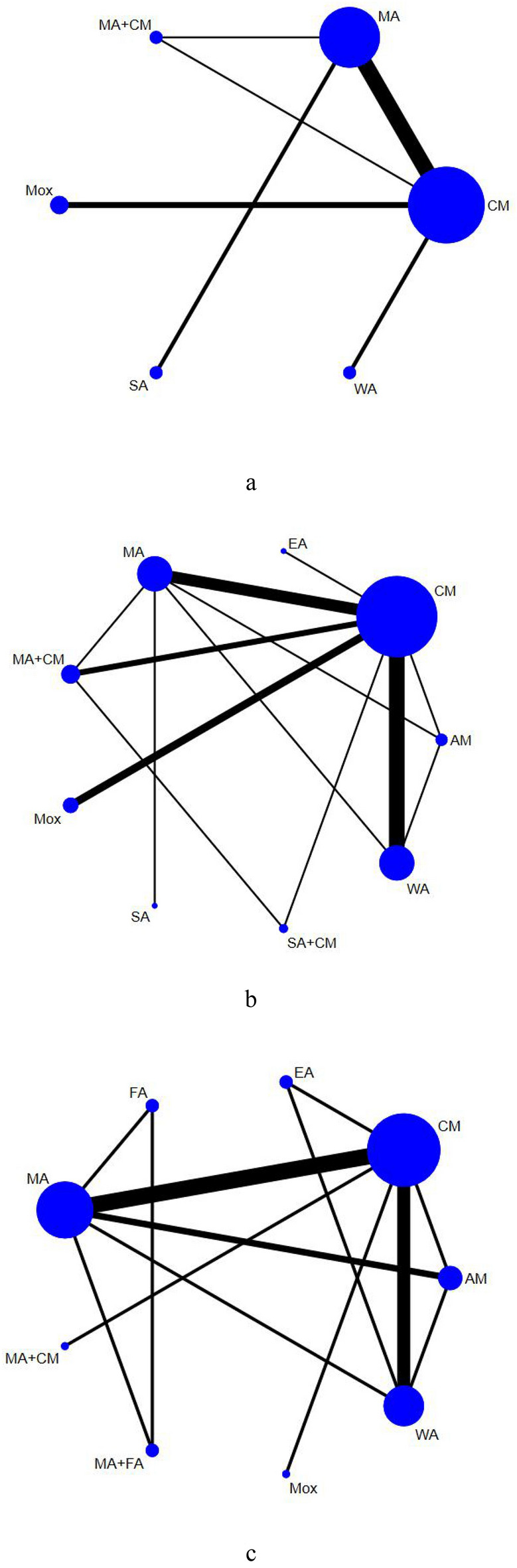
**a** The network graph of different interventions of TNSS; **b** The network graph of different interventions of reduction in RQLQ; **c** The network graph of different interventions of the Ig E

In Fig. 3, the thickness of the line is positively correlated with the two intervention methods, whereas the size of the points is proportional to the weight of the sample size in the intervention. A total of 15 studies covering 6 interventions and 995 participants were merged for meta-analysis of the reduction in TNSS (Fig. 3a). Reduction of RQLQ was reported in 24 RCTs including 2,080 patients and 9 methods (Fig. 3b), whereas reduction of Ig E was revealed in 17 studies involving 1,498 participants and 9 therapies (Fig. 3c).

#### Evaluating statistical inconsistency

All local inconsistency tests were performed with the node-splitting method. Results of the inconsistency test on the reduction of RQLQ and Ig E (P > 0.05) demonstrated that no significant difference existed between direct and indirect comparisons. Furthermore, no inconsistency of the model was found when the node-splitting method was used in TNSS, thus we selected the consistency model.

#### Reduction in TNSS

**Fig. 4:**
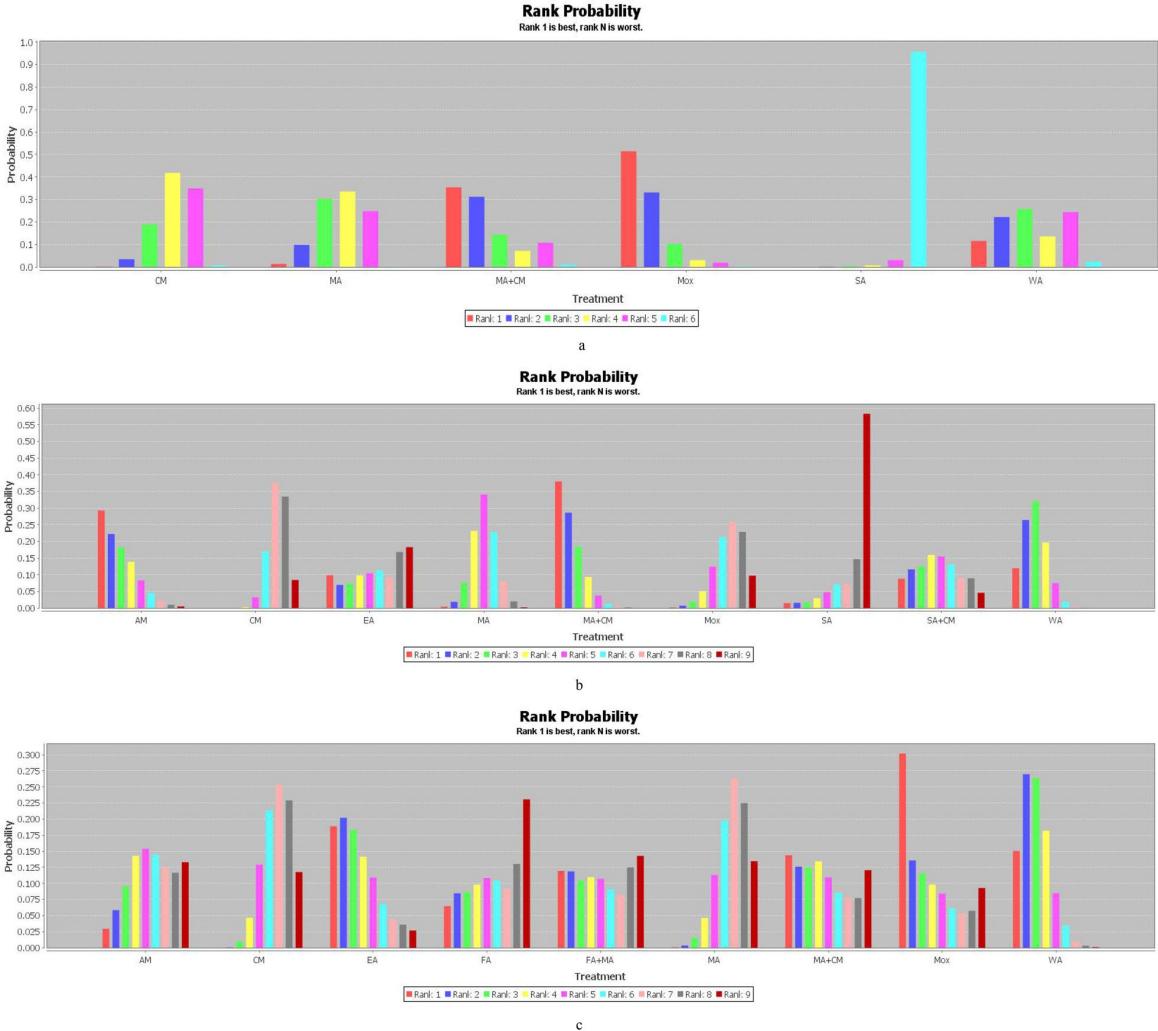
**a** The figure of ranking probability of reduction in TNSS; **b** The figure of ranking probability of reduction in RQLQ; **c** The figure of ranking probability of the change of Ig E

We conducted a network graph between the 6 treatments through STATA 15.0 (Fig. 3a). According to the PSRF results (value close to 1, Additional file 1: Appendix S1), we performed the network meta-analysis using the consistency model, and the figure of ranking probability was generated (Fig. 4a). Based on Fig. 4a, MA + CM, Mox, and WA treatments ranked the top 3 in this study. The Mox was recommended as the most effective intervention in reducing nasal symptoms out of the 6 treatments in this study. As highlighted in Table 6, 5 interventions (MA + CM, Mox, WA, MA, CM) were significantly more effective than SA.

**Table 6:** The results of network meta-analysis of reduction of TNSS

Mox					
0.80 (−6.05, 7.76)	MA+CM				
*3.00 (−1.37, 7.49)*	*2.21 (−3.62, 7.87)*	MA			
*3.31 (0.45, 7.26)*	*2.55 (−3.09, 8.27)*	0.33 (−2.01, 2.68)	CM		
*2.42 (0.45, 7.27)*	1.63 (−5.76, 9.27)	−0.62 (−5.86, 4.64)	0.95 (−3.85, 5.69)	WA	
*9.31 (2.95, 16.07)*	*8.52 (1.41, 16.35)*	*6.32 (1.54, 11.43)*	*5.97 (0.76, 11.56)*	*6.93 (−0.12, 14.16)*	SA

Italic values indicate significant difference

#### Reduction in RQLQ

Using STATA 15.0, we generated a network plot between the 9 interventions (Fig. 3b). Based on the PSRF (value close to 1, Additional file 1: Appendix S2 (a)) and P-values (Additional file 1: Appendix S2 (b)), Bayesian network meta-analysis was performed using the consistency model. The ranking probability figure was also generated (Fig. 4b). MA + CM, AM, and WA therapies ranked the top 3 in our study (Fig. 4b). The MA + CM was recommended as the most effective intervention in improving the quality of life based on the 9 treatments from the Bayesian meta-analysis. Moreover, 8 interventions (MA + CM, AM, WA, SA + CM, MA, EA, Mox, CM) were significantly more effective than SA (Table 7)

**Table 7:** The results of network meta-analysis of quality of life (RQLQ)

MA+CM								
*3.62 (−11.48, 19.07)*	WA							
*2.15 (−17.89, 23.24)*	1.41 (−15.90, 18.27)	AM						
*9.65 (−10.86, 29.68)*	*6.13 (−16.20, 27.73)*	*7.63 (−19.18, 33.36)*	SA+CM					
*11.07 (−3.46, 26.04)*	*7.54 (−3.86, 19.16)*	*9.09 (−8.51, 26.08)*	1.46 (−20.01, 23.30)	MA				
*14.75 (−15.74, 44.58)*	*11.15 (−17.73, 39.24)*	*12.42 (−19.01, 44.06)*	*4.96 (−28.87, 39.14)*	*3.45 (−25.30, 32.29)*	*EA*			
*17.36 (0.51, 34.82)*	*13.83 (−0.15, 27.85)*	*15.19 (−5.28, 35.01)*	*7.78 (−15.34, 31.51)*	*6.20 (−8.19, 20.86)*	*2.64 (−26.75, 32.19)*	Mox		
*18.80 (6.31, 32.02)*	*15.32 (7.19, 23.62)*	*16.71 (0.24, 32.65)*	*9.24 (−10.79, 30.01)*	*7.72 (−1.14, 16.76)*	*4.15 (−22.50, 31.49)*	1.52 (−9.92, 12.92)	CM	
*26.53 (−2.62, 54.85)*	*22.92 (−4.16, 49.63)*	*24.43 (−6.30, 53.77)*	*16.86 (−16.10, 49.79)*	*15.55 (−9.59, 39.13)*	*11.97 (−25.96, 48.87)*	*9.30 (−20.18, 36.80)*	*7.81 (−19.31, 33.01)*	SA

Italic values indicate significant difference

*MA* manual acupuncture, *EA* electroacupuncture, *WA* warm acupuncture, *Mox* moxibustion, *AM* Acupuncture-Moxibustion, *SA* sham acupuncture, *CM* conventional medicine

#### Reduction in Ig E

Using STATA 15.0, we generated a network graph between the 9 therapies (Fig. 3c). According to the PSRF results (value close to 1, Additional file 1: Appendix S3 (a)) and P-values (Additional file 1: Appendix S3 (b)), we performed the Bayesian analysis via the consistency model, and the figure of ranking probability was generated (Fig. 4c). Mox, EA, and WA methods ranked the top 3 in the study (Fig. 4c). Moxibustion was recommended as the most effective intervention in changing the level of Ig E of the 9 treatments. As shown in Table 8, 7 interventions (Mox, EA, WA, MA + CM, FA + MA, FA, AM) were significantly more effective than CM, meanwhile, compared with CM, MA has little effect in reduction of Ig E.

**Table 8:** The results of network meta-analysis of Ig E

WA								
*3.11 (−92.21, 98.28)*	Mox							
*3.61 (−52.13, 62.15)*	0.51 (−105.71, 106.01)	EA						
*16.89 (−66.06, 104.69)*	*13.57 (−101.21, 135.68)*	*13.66 (−81.95, 112.02)*	MA+CM					
*23.73 (−65.51, 118.96)*	*20.30 (−101.69, 146.41)*	*19.93 (−83.46, 124.12)*	*7.14 (−114.30, 125.63)*	FA+MA				
*30.35 (−26.32, 92.74)*	*27.35 (−76.58, 133.25)*	*26.19 (−49.60, 106.14)*	*12.78 (−81.05, 107.77)*	*6.63 (−91.93, 106.78)*	AM			
*33.48 (−55.68, 127.08)*	*30.54 (−92.75, 155.05)*	*30.32 (−73.22, 134.01)*	*16.50 (−100.39, 133.05)*	*10.19 (−69.46, 90.97)*	*3.98 (−95.17, 99.06)*	FA		
*40.43 (7.26, 76.07)*	*37.74 (−50.49, 126.01)*	*36.78 (−22.30, 93.11)*	*23.42 (−55.38, 100.04)*	*16.94 (−72.87, 102.00)*	*10.30 (−46.19, 62.39)*	*6.74 (−79.89, 91.96)*	CM	
*41.25 (−1.79, 87.41)*	*38.36 (−55.33, 131.83)*	*37.80 (−28.52, 101.34)*	*24.34 (−61.11, 108.24)*	*17.61 (−64.84, 97.82)*	*11.12 (−45.79, 64.63)*	*8.17 (−74.48, 88.90)*	0.82 (−30.25, 32.96)	MA

Italic values indicate significant difference

### Safety

Notably, 16 RCTs [41, 44, 45, 50, 53, 55, 57, 60, 61, 64–66, 68, 71, 73, 74], with 1,201 patients described information about safety. However, there were few adverse events associated with interventions (Table 9). The therapies included MA, WA, SA, CM. Acupuncture caused minor comfort, pain, headache, and skin trauma. Besides, CM caused and lethargy, headache, stomachache, and thirst. These AEs were acceptable, though no severe AEs occurred.

**Table 9:** Adverse events in included studies

Interventions	Sample size	Study	Results
MA	18	Lan 2010 [73]	A: 1 case of inconsequential bleeding
	42	Xue 2008 [74]	A: 11 cases of minor comfort, 1 case of headache, 1 case of dizziness
	32	Yu 2015 [64]	A: 2 cases with pain
WA	105	Sun 2020 [41]	A: 1 cases of dizziness, 2 cases fear acupuncture and moxibustion
	30	Zhang 2019 [44]	A: 1 case with dizziness, 1 case with blister
	49	Gao 2019 [45]	A: 3 cases of skin trauma, 2 cases of itchiness
	27	Wang 2013 [71]	A: 1case of inconsequential bleeding
SA	18	Lan 2010 [73]	B: 1 case of dizziness
	38	Xue 2008 [74]	B: 8 cases of minor comfortable, 2 cases of headache
CM	105	Sun 2020 [41]	B: 1 case of headache, 1 case of stomachache, 1 case of thirst
	32	Yu 2015 [64]	B: 2 cases with drowsiness

*MA* manual acupuncture, *WA* warm acupuncture, *SA* sham acupuncture, *CM* conventional medicine

### Heterogeneity

We conducted a sensitivity analysis using Revman to assess the stability and reliability of the joint meta-analysis results. Consequently, we found that our meta-analysis results were relatively stable. We appraised some factors, such as acupuncture methods, acupoints, duration of treatment among others to be different, which may cause high clinical heterogeneity.

### Publication bias

**Fig. 5:**
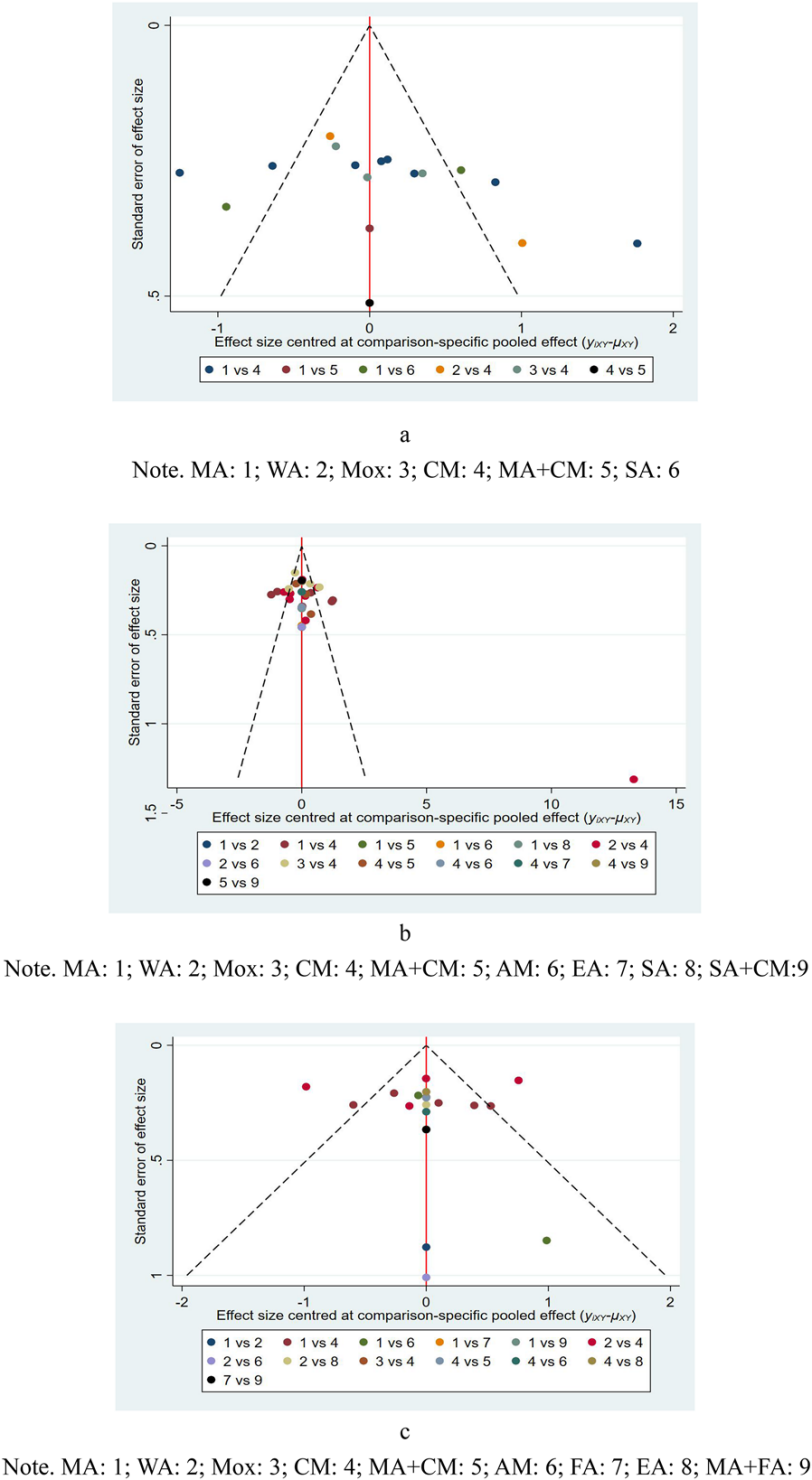
**a** Funnel plot for the network meta-analysis of reduction in TNSS; **b** Funnel plot for the network meta-analysis of reduction in RQLQ; **c** Funnel plot for the network meta-analysis of change of Ig E

The publication bias was evaluated by comparing the symmetry of the funnel plot. As shown in Figures in Fig. 5 for comparison-adjusted funnel graphs, the funnel graph illustrated that most of the RCTs were roughly symmetrically distributed on both sides of the midline. This demonstrated the decreased likelihood of small sample effects. However, there was no strong and powerful evidence of these small study effects across the outcomes.

## Discussion

Numerous studies have described the efficacy and safety of acupuncture for treating AR, a dominant disease across the globe. Despite a diverse number of acupuncture methods currently being applied, these methods have not been standardized. Consequently, clinicians are forced to combine several acupuncture-based methods, which is time-consuming and associated with high financial costs as well as wastage of medical resources.

In this systematic review, upon comparing the efficacy of different acupuncture interventions for AR, we yielded reliable results [77, 78]. For instance, Mox and MA + CM showed highly statistically significant differences in improving TNSS compared to CM when we conducted a pairwise meta-analysis. Moreover, the network Bayesian meta-analysis indicated Mox as the optimal therapy in reducing TNSS. When MA was integrated with CM, a higher reduction in the quality of life was realized compared with MA, CM, and SA + MA. MA and SA displayed significant differences, while SA + CM was more effective than CM. Both AM and WA showed significant differences with CM. Moreover, the MA plus CM was implicated as the most effective therapy in the reduction of RQLQ. Regarding the IgE, we found significant differences between MA plus CM in pairwise meta-analysis. Additionally, the Mox was indicated as the most effective treatment that changes the IgE content. Moreover, 16 RCTs (41.03%) reported safety in adopting these interventions. However, only 7 RCTs about acupuncture were associated with adverse events (minor comfort, pain, skin trauma, etc.) which were acceptable, though no severe adverse events occurred.

This systematic review has some limitations. Firstly, we purposed to reduce bias, however, it was unclear whether other potential studies were included. Most of the included RCTs were investigated in China, and the search language was limited to Chinese/English. Thus, this could potentially cause bias. Secondly, we, in most cases got 1 or 2 small trials to compare the effect of acupuncture methods, this could lead to insufficient statistical efficiency. Thirdly, some acupuncture studies had a challenge in determining the risk of bias. Therefore, researchers should be attempted to minimize bias. Additionally, acupuncture may result in numerous factors, such as the selection of acupoint, the treatment duration, among others. Meanwhile, the CM has differences in type, dosage, dosage forms which might cause heterogeneity. Moreover, most of the included trials [38, 47, 48, 51, 55, 60, 62] had a short treatment duration and lacked follow-up, which is still insufficient to illustrate the long-term effect of acupuncture.

Several previous trials demonstrated the clinical efficacy and safety of acupuncture for AR. However, numerous outcomes of the trials were dependent on the subjective records and feelings of these participants. Besides, because of the various interventions of acupuncture, inconsistencies occur in the selection of acupoint, duration, and frequency of treatment, among others. These factors may cause heterogeneity. Therefore, future research should be geared towards standardizing and generalizing acupuncture methods, acupoint, duration, and frequency of AR treatment. Moreover, since the methodological quality in our study was low to moderate, a well-designed RCTs should be implemented with the Consolidated Standards of Reporting Trials (CONSORT), Standards for Reporting Interventions in Clinical Trials of Acupuncture (STRICTA), Standards for Reporting Interventions in Clinical Trials of Moxibustion (STRICTOM) and Cochrane Handbook for Systematic Reviews of Interventions [79–81] to command the quality of future studies [82, 83]. Moreover, we suggest that protocols should be registered. The methodological quality of the included trials was not satisfactory, thus more precise and accuracy designed, generated, and published RCTs are warranted. Of note, we also wish future scholars to select outcome measures based on international consensus.

## Conclusion

This work identifies acupuncture as one of several effective therapies for AR. MA + CM/Mox may effectively improve AR symptoms and quality of life as demonstrated in pairwise and Bayesian network meta-analyses. Meanwhile, Mox was regarded as the most effective therapy that changes the IgE content from recently evidences. However, insufficient clinical evidence is presently available to guide on the selection of the acupoints, duration of treatment among others. Meanwhile, the overall quality of these included RCTs were mainly ranked as moderate. Therefore, lots of high-quality RCTs are required to validate the above-presented findings.

## Supplementary information

**Additional file 1: Appendix S1.** The PSRF value of reduction in TNSS. **Appendix S2.** (a) The PSRF value of reduction in RQLQ. (b) Node-splitting test result of reduction in RQLQ. **Appendix S3.** (a) The PSRF value of reduction in Ig E. (b) Node-splitting test result of reduction in Ig E.

## Abbreviations

AR: Allergic rhinitis
IgE: Immunoglobulin E
WOS: Web of science
CNKI: China National Knowledge Infrastructure
VIP: China Science and Technology Journal Database
CBM: Chinese Biomedical Literature Database
WHO ICTRP: World Health Organization International Clinical Trials Registry Platform
ChiCTR: Chinese Clinical Trial Register
AMED: Allied and Complementary Medicine Database
ADDIS: Aggregate Data Drug Information System
NMA: Network meta-analysis
ICC: Intra-class correlation coefficient
TNSS: Total nasal symptom score
RQLQ: Rhinoconjunctivitis quality of life questionnaire
MA: Manual acupuncture
SA: Sham acupuncture
CM: Conventional medicine
RCTs: Randomized controlled trials
Mox: Moxibustion
EA: Electronic acupuncture
WA: Warm acupuncture
AM: Acupuncture-moxibustion
FA: Fire acupuncture
PRISMA: Preferred Reporting Items for Systematic Reviews and Meta-analyses
AE: Adverse event
CI: Confidence interval
SMD: Standard mean difference
MCMC: Markov Chain Monte Carlo
PSRF: Potential scale reduced factor
Revman: Review manager
WF: Wanfang Database
