# Supplementary material for: Acupuncture methods for allergic rhinitis: a systematic review and bayesian meta-analysis of randomized controlled trials
Source: Chin Med. 2020 Oct 12;15:109. doi: 10.1186/s13020-020-00389-9 (PMC7552548; doi:10.1186/s13020-020-00389-9)
Supplement: Supplementary file 1 — Additional file 1: Appendix S1. The PSRF value of reduction in TNSS. Appendix S2. (a) The PSRF value of reduction in RQLQ. (b) Node-splitting test result of reduction in RQLQ. Appendix S3. (a) The PSRF value of reduction in Ig E. (b) Node-splitting test result of reduction in Ig E. [file 13020_2020_389_MOESM1_ESM.pdf]

Appendix 1.

The PSRF value of reduction in TNSS

Parameter	PSRF
d.CM.MA	1.00
d.CM.MA+CM	1.00
d.CM.Mox	1.00
d.CM.WA	1.00
d.MA.SA	1.00
sd.d	1.00

Note: MA: manual acupuncture; WA: warm acupuncture; Mox: moxibustion; SA: sham acupuncture; CM: conventional medicine

Appendix 2.

(a) The PSRF value of reduction in RQLQ.

Parameter	PSRF
d.CM.AM	1.00
d.CM.EA	1.00
d.CM.MA	1.00
d.CM.MA+CM	1.00
d.CM.Mox	1.00
d.CM.SA+CM	1.00
d.CM.WA	1.00
d.MA.SA	1.00
sd.d	1.00

(b) Node-splitting test result of reduction in RQLQ.

Name	Direct Effect	Indirect Effect	Overall	P-Value
AM, CM	-26.46 (-49.46, -3.20)	-8.13 (-30.54, 13.96)	-16.71 (-32.65, -0.24)	0.24
AM, MA	-3.24 (-29.32, 22.66)	-9.94 (-30.39, 10.18)	-9.09 (-26.08, 8.51)	0.67
AM, WA	6.57 (-17.34, 31.49)	-5.03 (-25.09, 15.59)	-1.41 (-18.27, 15.90)	0.44
CM, MA	8.30 (-1.96, 18.16)	9.81 (-14.48, 35.45)	7.72 (-1.14, 16.76)	0.91
CM, WA	14.32 (5.57, 23.30)	23.13 (-1.13, 47.88)	15.32 (7.19, 23.62)	0.48
MA, MA+CM	18.17 (-5.78, 41.24)	4.41 (-14.15, 22.73)	11.07 (-3.46, 26.04)	0.34
MA, WA	11.51 (-13.56, 36.86)	5.75 (-6.68, 18.76)	7.54 (-3.86, 19.16)	0.67

Note: MA: manual acupuncture; EA: electroacupuncture; WA: warm acupuncture; Mox: moxibustion; AM: Acupuncture-Moxibustion; SA: sham acupuncture; CM: conventional medicine

Appendix 3.

(a) The PSRF value of reduction in Ig E.

Parameter	PSRF
d.CM.AM	1.00
d.CM.EA	1.00
d.CM.MA	1.00
d.CM.MA+CM	1.00
d.CM.Mox	1.00
d.CM.WA	1.00
d.MA.FA	1.00
d.MA.FA+MA	1.00
sd.d	1.00

(b) Node-splitting test result of reduction in Ig E.

Name	Direct Effect	Indirect Effect	Overall	P-Value
AM, CM	-24.09 (-105.12, 58.80)	3.04 (-70.76, 78.38)	-10.30 (-62.39, 46.19)	0.61
AM, MA	-23.08 (-94.04, 54.45)	3.36 (-67.12, 82.85)	-11.12 (-64.63, 45.79)	0.56
AM, WA	80.99 (-12.70, 181.90)	11.19 (-52.66, 79.27)	30.35 (-26.32, 92.74)	0.18
CM, EA	28.68 (-57.91, 115.41)	44.80 (-44.41, 135.86)	36.78 (-22.30, 93.11)	0.79
CM, MA	2.39 (-34.04, 39.29)	-24.56 (-108.61, 59.61)	-0.82 (-32.96, 30.25)	0.53
CM, WA	33.99 (-6.49, 73.94)	63.58 (-8.82, 137.17)	40.43 (7.26, 76.07)	0.46
EA, WA	-2.70 (-88.43, 79.79)	12.88 (-80.30, 106.86)	3.61 (-52.13, 62.15)	0.78
MA, WA	76.43 (-23.28, 171.17)	29.61 (-19.01, 77.18)	41.25 (-1.79, 87.41)	0.37

Note: MA: manual acupuncture; EA: electroacupuncture; WA: warm acupuncture; FA: fire acupuncture; Mox: moxibustion; AM: Acupuncture-Moxibustion; CM: conventional medicine
